# Local variability in *Pfhrp2* and *Pfhrp3* gene deletions prevalence: Approaches, estimation methods, and reporting - A systematic review and meta-analysis

**DOI:** 10.5281/zenodo.20137112

**Published:** 2026-05-12

**Authors:** Moussa B. Kanoute, Amadou Niangaly, Etienne Guirou, Entoine Dara, Issaka Sagara, Abdoulaye Djimde

**Affiliations:** 1 Parasites and Microbes Research & Training Center, Faculty of Pharmacy, University of Sciences, Techniques and Technologies of Bamako, Mali.

## Abstract

**Background:**

Currently, the widely used antigen-based malaria rapid diagnostic tests (RDTs) in sub-Saharan Africa primarily detect *Plasmodium falciparum* histidine-rich protein 2 (PfHRP2). However, their diagnostic accuracy is being compromised by the increasing report of evolving parasite strains with deletion of *pfhrp2* and *pfhrp3* genes in malaria endemic regions. Therefore, elucidating local heterogeneity and addressing methodological variability are critical for guiding diagnostic strategies.

**Materials and Methods:**

We systematically searched PubMed and Google Scholar for studies published between 2015 to 2024 reporting the prevalence of *pfhrp2* and/or *pfhrp3* gene deletions. Eligible studies utilised molecular confirmation methods and provided regional or national prevalence estimates. A random-effects meta-analysis was conducted to estimate pooled prevalence and evaluate heterogeneity. Additionally, study methodologies and compliance with World Health Organization (WHO) guidelines were evaluated.

**Results:**

Forty-three studies from 60 sampling sites across Africa, the Americas, and Asia were included. The highest pooled prevalence of *pfhrp2* deletion was observed in the Americas (35%, 95% CI: 17–55%), followed by Africa (9%, 95% CI: 4–17%) and Asia (3%, 95% CI: 2–4%). Markedly elevated high local deletion rates (>80%) were reported in specific areas of Ethiopia, Eritrea, Peru, and Colombia. Notably, substantial intra-national variability was evident, particularly within Central Africa and South America. Most studies (81%) complied with WHO-recommended protocols for deletion surveillance. Delays of 5-10 yrs between sample collection and publication were common, raising concerns about data timeliness although methodological heterogeneity in sampling strategies, molecular detection methods, and study design contributed to variability in reported estimates.

**Conclusions:**

High prevalence of *pfhrp2/3* gene deletions compromises the effectiveness of PfHRP2-based RDTs in several regions. Countries with a prevalence rate of 5% and above are strongly encouraged to transition to alternative antigen-based RDTs, such as pLDH-based or combination tests. Standardising molecular surveillance methods, expediting data reporting and ensuring alignment with WHO protocols are critical to inform and sustain an evidence-based diagnostic framework tailored to region-specific malaria control policies.

## Introduction

Global efforts in malaria control and elimination were able to preclude approximately 2.2 billion cases and 12.7 million deaths since 2000 [1]. Despite the encouraging progress, malaria continues to affect hundreds of millions of people, with sub-Saharan Africa bearing the largest burden. In 2023, an estimated 263 million cases of malaria were reported that caused 597,000 deaths globally reflecting an increase of 11 million cases compared to 2022 with a slight decline in deaths [1, 2].

The World Health Organization (WHO) African Region remains the most affected, accounting for 94% of global cases and 95% of malaria-related deaths in 2023. Four countries, Nigeria (30.9%), the Democratic Republic of Congo (11.3%), Niger (5.9%), and Tanzania (4.3%) together contribute to more than half of all malaria deaths, with *Plasmodium falciparum* as the dominant species [3]. Moreover, two-thirds of the global burden is concentrated in eleven African countries: Burkina Faso, Cameroon, the Democratic Republic of Congo, Ghana, Mali, Mozambique, Niger, Nigeria, Sudan, Tanzania, and Uganda [3]. As of late 2024, 44 countries and one territory had been certified malaria-free by WHO, and 25 endemic countries reported fewer than 10 cases annually [4].

Current malaria control strategies include the use of long-lasting insecticide-treated nets (LLINs), indoor residual spraying (IRS), intermittent preventive treatment in pregnancy (IPTp)[5], seasonal malaria chemoprevention (SMC) in children, and artemisinin-based combination therapies (ACTs). Additionally, the two licensed vaccines (RTS,S/AS01 and R21/Matrix-M) are being gradually implemented several countries [4, 6]. Currently, WHO recommends that all suspected malaria cases be confirmed by parasitological diagnostics. Given the limited availability of trained microscopists in many settings, rapid diagnostic tests (RDTs) have emerged as the primary tool for malaria detection, accounting for over 75% of tests in Africa by 2017 and 31.2% of malaria diagnoses in Europe also rely on RDTs or in combination with microscopy [7].

Extensively used RDTs detect the *Plasmodium falciparum* histidine-rich protein 2 (PfHRP2) antigen. However, reports of *P. falciparum* strains with deletions in the *pfhrp2* and *pfhrp3* genes have raised significant concerns. *Pfhrp2* and *pfhrp3* gene deletions are highly prevalent in the Horn of Africa, particularly in Eritrea, where 80.8% and 92.3% of parasites carry *pfhrp2* and *pfhrp3* deletions, respectively [8, 9]. Similar patterns have also been reported in parts of the Amazon and Asia [10–12]. WHO recommends reconsideration of HRP2-based RDT use only when *pfhrp2/3* gene deletions are demonstrated to cause clinically significant false-negative HRP2-RDT results and when their prevalence exceeds the 5% at the lower bound of the 95% confidence intervals (CIs) in the target population [13]. Studies conducted in Africa report a highly variable prevalence of single and double deletions of the *pfhrp2* gene, ranging from 0.4% [14] in some regions to 60% [12] in others, with extreme values of 80% [15] in certain areas of the Horn of Africa. In West Africa, rates where 2% in Mali and 4.4% in Burkina Faso, highlighting urgent public health concerns given the reliance on PfHRP2-based RDTs in sub-Saharan Africa [16–18]. However, methodological differences across studies, such as sample size, participant clinical status, geographic scope, and detection protocols limit accurate comparability of prevalence estimates and may misguide policy decisions. To harmonise data, the WHO Global Malaria Programme has established standardised protocols for investigating and reporting *pfhrp2* and *pfhrp3* gene deletions [2].

Thus, this review aims to assess the local variability in *pfhrp2/pfhrp3* gene deletions prevalence between 2015 and 2024 and to evaluate the methodologies and reporting approaches employed in published studies across continents.

## Materials and Methods

A systematic literature search was conducted using PubMed and Google Scholar to identify peer-reviewed articles published between May 2015 and December 2024.

The search strategy combined Medical Subject Headings (MeSH) and free-text keywords related to *Plasmodium falciparum* and gene deletions. The search terms included: ("*Plasmodium falciparum*" OR "*P. falciparum*") AND ("*pfhrp2* deletion" OR "histidine rich protein 2 deletion" OR "HRP2 gene deletion") OR ("*pfhrp3* deletion" OR "HRP3 gene deletion") AND ("prevalence" OR "frequency") AND ("diagnostic" OR "RDT" OR "rapid diagnostic test") AND ("Africa" OR "Asia" OR "South America" OR "Amazon" OR "Horn of Africa”).

In Google Scholar, simplified combinations of the keywords were applied to capture grey literature and additional relevant sources. Boolean operators AND and OR were used to combine concepts. Filters were applied to restrict the search to: English-language articles, Original research, Human studies. Additional references were identified by screening the bibliographies of relevant publications to ensure comprehensive coverage of available evidence. Articles were included if they met the following criteria: original research reporting the prevalence of *pfhrp2* and/or *pfhrp3* deletions, studies providing region-specific data, and a detailed methodological description for gene deletion detection. Exclusion criteria comprised review articles, conference abstracts, editorials, correspondence, non-human studies were excluded, reports not focused on *P. falciparum*, and articles published outside the target time frame.

**Figure 1:**
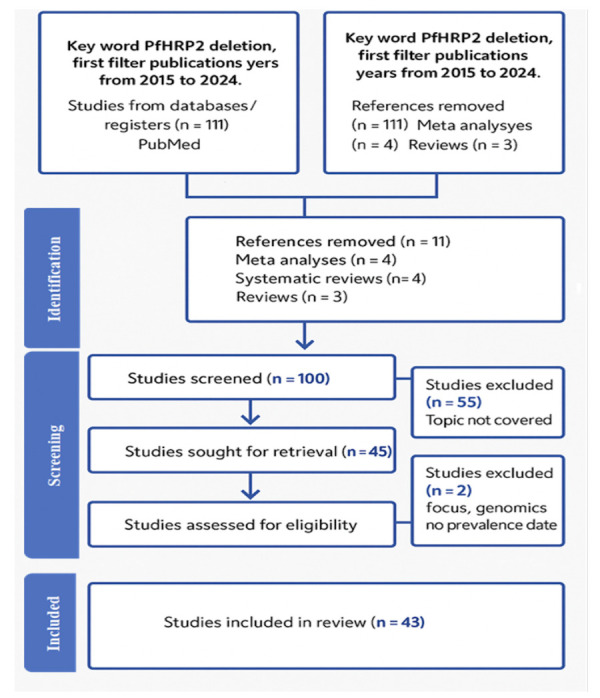
Stepwise process of article selection.

Study quality was assessed for data extraction in Covidence platform (version 2025) using criteria adapted from the Joanna Briggs Institute (JBI) checklist for prevalence studies [19], evaluating sample representativeness, sample size, data reliability, and diagnostic clarity. Two reviewers assessed each study independently, resolving disagreements by consensus. Studies were not excluded based on quality, but results were interpreted accordingly. A PRISMA flow diagram [20] (Figure 1) was generated to illustrate the study selection process.

Studies were categorized by geography (continent, subregion, country), design, laboratory methods, clinical population (symptomatic vs. asymptomatic), and the extent of the deletion (*pfhrp2*-only or dual including *pfhrp3* and flanking genes).

Data exported from Covidence in CSV format were analysed in R Studio (version 2024.12.0) using a random-effects meta-analysis. Pooled prevalence and 95% CIs were calculated, with heterogeneity

assessed using Cochran’s Q statistic and quantified with the I^2^index. Subgroup analyses by deletion type, molecular method, and region explored residual heterogeneity, while sensitivity analyses evaluated the impact of individual studies on over all estimates [21].

## Results

Among the 111 articles initially identified, 43 fulfilled the inclusion criteria and were incorporated into the final analysis [8-12,14,15,22-56]. The majority provided information on both *pfhrp2* and *pfhrp3* gene deletions, together with detailed descriptions of laboratory methods, sampling periods, and regional prevalence estimates. Of these 43 studies, 30 originated from African countries, the remaining studies included nine from the Americas and four from Asian countries (Table 1). Blood samples analysed in these studies were collected from symptomatic and/or asymptomatic patients between 2000 and 2024. A total of 18543 samples with *P. falciparum* infection were tested for *pfhrp2* and *pfhrp3* gene deletions.

**Table 1:** List of articles included in the study.

#	Reference	Sample Size	Symptomatic	Country / Region	Year	Compliance
1	[22]	100	Yes	Guyana	2015	Compliant
2	[10]	115	Yes	Colombia	2015	Compliant
3	[23]	784	No	Ghana	2016	Non-Compliant
4	[24]	1521	Yes	India	2016	Compliant
5	[25]	365	Yes	Colombia (Amazon)	2016	Compliant
6	[26]	783	Both	DRC	2017	Compliant
7	[27]	198	Yes	Brazil	2017	Compliant
8	[28]	144	ND	Eritrea	2017	Compliant
9	[29]	274	No	Kenya	2017	Non-Compliant
10	[30]	9124	Both	Mozambique	2017	Compliant
11	[8]	50	Yes	Eritrea	2018	Partially
12	[31]	128	Yes	Guatemala	2018	Compliant
13	[12]	1058	Yes	India (Odisha)	2018	Compliant
14	[32]	511	Yes	Nigeria	2019	Compliant
15	[33]	911	Both	Ghana	2019	Compliant
16	[34]	1724	ND	Equatorial Guinea	2020	Partially
17	[35]	138	ND	Mekong Sub-region	2020	Partially
18	[36]	300	Yes	Uganda	2020	Compliant
19	[15]	378	Yes	Djibouti	2020	Compliant
20	[37]	7543	No	Tanzania (mainland)	2020	Non-Compliant
21	[14]	64	Yes	Ethiopia	2020	Partially
22	[55]	192	Yes	Brazil (Acre State)	2020	Compliant
23	[38]	3516	Both	Uganda	2021	Compliant
24	[39]	210	Yes	Nigeria	2021	Compliant
25	[40]	218	Yes	Ethiopia (Assosa Zone)	2021	Compliant
26	[9]	12572	Both	Ethiopia	2021	Compliant
27	[41]	1109	Yes	DRC	2021	Compliant
28	[42]	250	Yes	India	2022	Compliant
29	[47]	2302	Yes	Ethiopia	2022	Compliant
30	[44]	998	Both	Tanzania	2022	Compliant
31	[45]	634	Both	DRC	2022	Compliant
32	[46]	324	Yes	Cameroon	2022	Compliant
33	[43]	1002	Yes	Djibouti	2022	Compliant
34	[48]	354	Yes	Ethiopia (Amhara)	2022	Compliant
35	[11]	325	Yes	Peru	2022	Compliant
36	[49]	594	Yes	South Sudan	2023	Compliant
37	[50]	2037	Both	Gabon	2023	Compliant
38	[51]	1000	Yes	India (Kolkata)	2023	Compliant
39	[52]	2435	Yes	Uganda (North)	2024	Compliant
40	[56]	7863	Yes	Tanzania	2024	Compliant
41	[53]	354	Yes	South Africa	2024	Compliant
42	[17]	566	Both	Equatorial Guinea	2024	Compliant
43	[54]	82	Both	Brazil (Middle Rio Negro)	2024	Partially

Most of the studies we selected for this work (35, or 81%) comply with WHO recommendations, and most of them were published between 2020 and 2024, indicating a gradual adoption of WHO recommendations. Of the 43 articles included, 10 were published before 2018. Of these, 5 were partially compliant with WHO recommendations, representing 11.6% of all studies, while 3 (6%) did not comply with the guidelines for molecular surveillance of *pfhrp2/3* deletions (Table 2). These non-compliant studies predate the implementation of WHO Protocol No. 9. Conversely, in regions with high malaria transmission, such as sub-Saharan Africa and India, compliance levels were generally higher, suggesting better integration of WHO recommendations into national malaria control programmes [23].

**Table 2:** Compliance with WHO recommended criteria for monitoring *pfhrp2*/*pfhrp3* deletions.

WHO criterium (WHO guidelines)	Description	Compliance across studies	Common deviations observed
HRP2-RDT negative result	Sample must be negative by HRP2-based RDT before suspicion of deletion	Partial	Some studies screened samples regardless of RDT result
Microscopy or PfLDH confirmation	Sample must be positive by microscopy or PfLDH- based RDT	Partial	Absence of PfLDH testing; reliance on PCR only
Confirmation of *P. falciparum* mono-infection	Exclusion of mixed-species infections by PCR	Moderate	Species confirmation not systematically reported
Amplification of *pfhrp2* exon 1 and exon 2	Required for molecular confirmation of deletion	Moderate to high	Some studies targeted only one exon
Amplification of *pfhrp3* exon 1 and exon 2	Required to confirm *pfhrp3* deletion	Moderate	Incomplete reporting or selective testing
DNA quality control (MSP1/MSP2)	Verification of DNA integrity before declaring deletion	Low to moderate	Quality control genes not consistently amplified
Standardized reporting of methods	Clear documentation of diagnostic and molecular workflow	Variable	Incomplete methodological descriptions

### Annual prevalence of *pfhrp2* and *pfhrp3* deletions from 2015 to 2024

For all articles included in our review analysis, samples were collected between 2001 and 2022, depending on the article, and published between 2015 and 2024.

It should be noted that the types of samples used to estimate the prevalence of deletions varied from one study to another, which explains why not all the articles included in this review comply with WHO recommendations (Table 1). Some studies followed the guidelines defined by the WHO (Table 2) and focused on so-called discordant samples, i.e., those that were negative on the HRP2 rapid diagnostic test but positive on microscopy or the PfLDH test. Others, however, analysed all parasitaemia samples without considering the results of the rapid test. This distinction is essential because prevalence estimates based on discordant samples tend to be higher and are used specifically to assess the risk of false negatives associated with rapid tests based on HRP2 detection.

**Figure 2:**
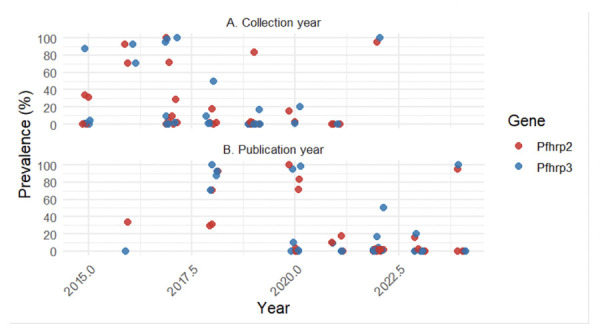
Prevalence of *pfhrp2* and *pfhrp*3 deletions from 2015 to 2024 by collection year (A) and publication year (B). Most data points originate from discordant HRP2-RDT–negative but microscopy/PfLDH-positive samples.

Figure 2A illustrates the temporal distribution of sample collection, showing a broad span from 2000 to 2022, with a dense cluster of samples between 2010 and 2018. Cases of very high prevalence (75–100%) appear for collection years 2016-2022, which may reflect emerging resistance hotspots during that period. Very high deletion prevalences (75–100%) are mostly observed in studies conducted between 2016 and 2022. However, these high values often originate from targeted investigations in suspected hotspots or from discordant-sample designs rather than from population-representative surveys. In contrast, most studies reporting general sampling approaches show prevalences in the 0–25% range, suggesting that deletions remain relatively low in many settings.

**Figure 3:**
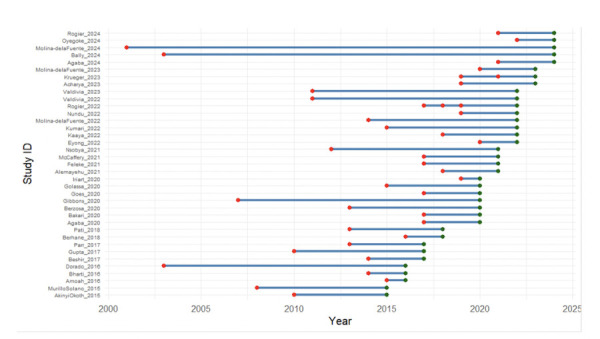
Time between sample collection and publication of articles, in years (red dot = collection year; green dot = publication year.

The *pfhrp2* and *pfhrp3* genes show relatively similar deletion patterns, although sometimes shifted, there is a clear temporal gap between the sample collection (Figure 2A) and the publication dates (Figure 2B). This lag can obscure the actual evolution of prevalence if not carefully considered. The temporal analysis highlights the delays between collection and publication, which vary considerably from one study to another, often with a median delay of 3 years (IQR: 2 to 5 years)(Figure 3). This delay significantly compromises the timeliness of surveillance data, as the reported prevalences may no longer reflect the current epidemiological situation at the time of publication. Given the dynamic nature of *pfhrp2/3* deletions under selective diagnostic pressure, these delays may lead to the use of outdated data for policy decisions. Some more recent studies [8,15,24,53] tend to reduce the gap between sampling and publication. This may reflect better study organisation or faster analysis methods. Others, however, have experienced delays of several years. This disparity may reflect differences in the complexity of the analyses, available resources, or publication priorities.

To ensure that diagnostic recommendations remain relevant in the face of evolving parasite populations, future surveillance efforts should prioritise strategies to shorten the interval between field sampling and dissemination of results, including using preprints, open-access databases, and realtime molecular surveillance platforms.

When interpreting the implications for RDT reliability, it is therefore essential to consider whether prevalence estimates are based on discordant samples or on broader surveillance designs, as these approaches answer different epidemiological and programmatic questions.

### Local variability in the prevalence of *pfhrp2* and *pfhrp3* deletions

In our local context, variability refers to differences in the prevalence of *pfhrp2/3* gene deletions within a given country between different regions or localities, within the same region, and sometimes even between neighbouring districts, study sites, or periods.

**Figure 4:**
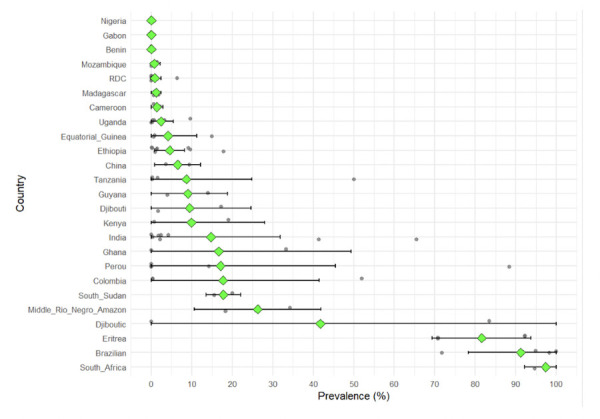
Between and within country variation of the PfHRP2/3 deletions prevalence. Grey dots are individual studies, green diamonds the mean, error bars show 95% CI.

Figure 4 shows interregional and intraregional variations in the prevalence of *pfhrp2* deletion, with some regions such as Eritrea, Brazil, and South Africa showing extremely high prevalence (>80%), while several other countries report intermediate levels (20-80%) and others consistently low levels (<10%). The errors indicate a high degree of uncertainty, reflecting local data variability or limited sample size.

A key finding is that marked within-country variability can occur even during overlapping surveillance periods, suggesting both spatial heterogeneity and temporal dynamics. In Ethiopia, prevalence estimates range from less than 5% in some districts to more than 40% in others, although they were collected during overlapping surveillance periods [9,43,57,58]. Similarly, Eritrea had a high overall prevalence, but with notable differences between sentinel sites. In Peru, Amazonian regions such as Loreto reported deletion rates above 40%, while coastal and Andean sites recorded much lower levels, sometimes below 5%. Colombia also showed intra-country heterogeneity, with some regions studied affected by moderate deletion prevalence, while prevalence rates above 50% were reported in other regions [9,14,43,48]. These patterns underscore that deletion prevalence is rarely uniform within national borders and that subnational data are essential for diagnostic policy decisions.

Beyond actual epidemiological variations, several methodological factors contribute to the differences observed between studies and may partly explain the heterogeneity observed within and between countries. The choice of molecular method (nested PCR, conventional PCR, multiplex PCR, or qPCR) strongly influenced estimates. Nested PCR, which is widely used, is prone to false negatives when parasitaemia is low or DNA quality is suboptimal: under these conditions, the absence of *pfhrp2/ pfhrp3* amplification can be confused with a deletion if internal controls (*msp1*, *msp2*, housekeeping genes) do not function properly. Conversely, approaches based on qPCR or multi-target tests are more reliable in cases of low parasitaemia, but their use remains highly variable across studies. Finally, differences in primers, target regions (exon 1 or exon 2), and detection criteria complicate the comparison of results and can generate artificial variability.

Polyclonal infections are also a determining factor in the detection of deletions, particularly in areas of moderate to high transmission where multiple clones are common. When deleted and non-deleted parasites coexist in the same individual, amplification methods tend to favour clone with the intact gene, which can mask deletions and lead to an underestimation of their prevalence. Conversely, in low-density mixed infections, random amplification failure can give the illusion of a deletion. None of the studies analysed considered the multiplicity of infections using genetic markers or specific approaches to identify mixed infections, which probably contribute to the discrepancies observed between sites and between survey periods.

Sampling strategies also directly shape estimates: Studies based on discordant samples, enriched with suspected deletions, automatically reported higher prevalences than non-selective surveys. Finally, the clinical status of participants, the level of transmission, and the sampling environment influence parasite density and infection multiplicity, thereby affecting the probability of detecting deletions.

### DNA Extraction

Commercial kits were mostly used for DNA extraction (Table 3) including Qiagen kit in 24 of the 43 studies, as well as Biotools, PureLink, and Zymo. A few studies used also the Chelex-100 and Saponin combination as well as phenol-chloroform DNA extraction methods.

**Table 3:** Between and within country variation of the PfHRP2/3 deletions prevalence. Grey dots are individual studies, green diamonds the mean, error bars show 95% CI.

DNA Extraction Method	Micro-satellite	Nested PCR	RT-qPCR	Total
Chelex-100 / Saponin method	2	9	2	13
DNA extraction kit (Biotools, Spain)	0	1	0	1
DNA kit (Qiagen)	1	14	9	24
Genomic purification	0	1	1	2
Phenol-chloroform extraction	0	0	1	1
PureLink DNA kit	0	1	0	1
Zymo Quick-DNA kit	0	1	0	1
Total	3	27	13	43

Nested-PCR was used by most of the study (27/43 studies) for the molecular detection of *pfhrp2* gene deletions followed by the RT-qPCR (13/43).

The recommended laboratory protocols for confirming deletions of the *pfhrp2* and *pfhrp3* genes in *P. falciparum* are clearly defined by the WHO [2]. and describe the essential diagnostic steps to ensure validity. For a sample to be considered suspicious for deletion, it must first be negative on the HRP2-based rapid diagnostic test (RDT) but positive on microscopy or an RDT targeting PfLDH. Next, a dried blood spot (DBS) is collected for molecular analysis. PCR tests are then performed to differentiate between *P. falciparum* mono-infection and mixed infections.

PCR for amplification of regions of exons 1 and 2 of the *pfhrp2* and *pfhrp3* genes is performed on samples confirmed as *P. falciparum*. The absence of amplification, only after successful amplification of quality control markers such as *msp1* and *msp2*, is considered evidence confirming gene deletion. Failure to verify DNA integrity can lead to false-positive deletions due to poor DNA quality or suboptimal PCR conditions.

However, several studies included in this review [12,17,27,30,44], did not consistently follow these quality control criteria, particularly the requirement that only samples that are negative for HRP2-RDT but positive for microscopy or PfLDH testing should be screened for deletions. This deviation compromises the comparability and reliability of the reported deletion rates. In addition, DNA extraction methods varied considerably between studies.

### Prevalence of pfhrp2 and pfhrp3 deletions in *P. falciparum* populations worldwide from 2015 to 2024 and study compliance with WHO pfhrp2 deletion protocol

**Figure 5:**
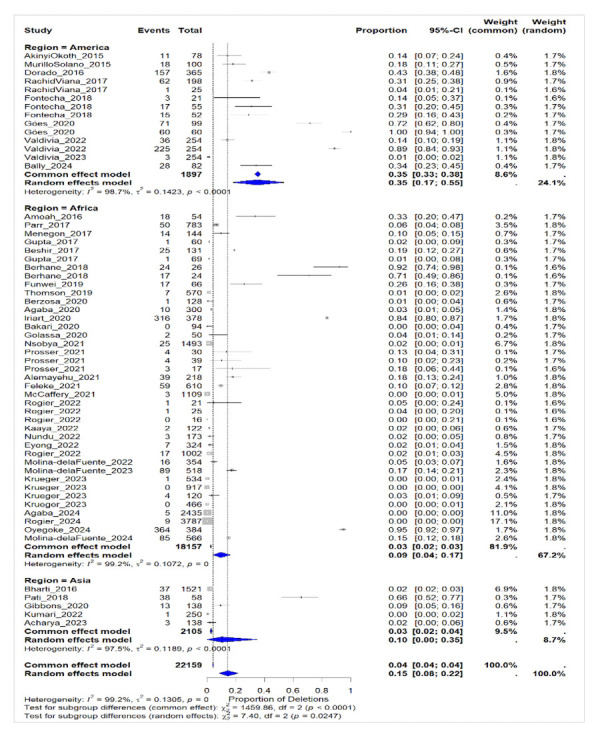
Forest plot showing the prevalence of *pfhrp2* deletions worldwide and by WHO region.

The global pooled prevalence of *pfhrp2* deletions was highest in the America at 35% (95% CI: 17–55%), followed by Africa (9%; 95% CI: 4–17%) and Asia (3%; 95% CI: 2–4%). A high level of statistical heterogeneity was observed across studies (I^2^= 99.2%) (Figure 5).

Some regions exhibited alarmingly high deletion rates, particularly the Horn of Africa: deletion rates in Eritrea 62.8% [47] and Ethiopia (Amhara) 51.4% [59]; Amazon Basin: >80% in localised areas of Peru ~70% [60], Colombia 38.5% [61], and Brazil Acre Stade, Amazon Basin 31.6% [54].

Deletions of the *pfhrp2* gene exhibit significant geographical variability. They are relatively high in South/Central America and the Horn of Africa, where they sometimes exceed 90%, seriously compromising the reliability of PfHRP2-based rapid diagnostic tests. In contrast, they remain generally low in Asia, where these tests remain relevant for malaria detection.

The very high heterogeneity observed among studies (I^2^> 95%) reflects major differences in epidemiological contexts, selection pressures related to RDTs, detection methods, and sample sizes. Statistical tests confirm significant differences between sub-regions. Despite the possible influence of small studies, sample sizes (<50 subjects), and publication bias, the robustness of the analysis is reinforced by the large total number of participants (n = 22,159) and the subgroup analysis. These results support the need to adapt diagnostic strategies in regions with a high prevalence of deletions, particularly in East Africa and the Americas.

## Discussion

This systematic review highlights significant geographical and temporal variability in the prevalence of *pfhrp2* and *pfhrp3* gene deletions in *P. falciparum* populations across endemic regions. Higher rates of *pfhrp2* and *pfhrp3* gene deletions (>80%) [8, 47] in the Horn of Africa and parts of the Amazon Basin pose serious challenges for the continued use of PfHRP2-based RDTs in these settings. The uneven distribution of gene deletions implies that current diagnostic strategies cannot be equally recommended for all malaria-endemic regions. In areas with low deletion prevalence (below 5%) HRP2-based RDTs remain a viable and cost-effective tool. However, in high-deletion zones, the use of these tests may lead to false negatives, missed diagnosis, risk of severe malaria, and increased transmission.

The high prevalence of *pfhrp2/3* gene deletions reported in Ethiopia has prompted national health authorities to revise their malaria diagnostic policy, replacing HRP2-based with pLDH-based RDTs [17]. However, in regions where high deletion prevalence has been documented, continued reliance on HRP2-based RDTs may lead to false-negative results, missed diagnoses, increased risk of severe disease, and sustained malaria transmission.

These programmatic responses illustrate the direct implications of molecular surveillance data for malaria control strategies. A major conclusion of this review is the significant heterogeneity observed between studies on *pfhrp2* and *pfhrp3* deletions. This variability partly reflects genuine epidemiological differences in parasite populations depending on geographical context, but it is also strongly influenced by the methodological approaches used.

Sampling strategies are one of the main sources of divergence. Some studies have targeted so-called ‘discordant’ samples, i.e., samples that are negative on HRP2-based RDTs but positive on microscopy or PfLDH tests, which intentionally increases the probability of identifying deletions [10, 22]. Conversely, other studies have relied on samples from unselected clinical populations or community surveys [37]. Such differences in sampling frameworks strongly influence the reported prevalence estimates and limit inter-study comparability.

In addition, there is considerable diversity in the molecular methods used to detect deletions. Nested PCR remains the most common technique, while RT-qPCR, which is more sensitive and allows relative quantification, remains less widely used due to financial and technical constraints. Variations in primer design, targeted genomic regions, and amplification conditions can also affect detection, particularly in samples with low parasite density. Together, these factors highlight the need for methodological harmonisation to improve the accuracy of estimates and the comparability of results at the regional and global levels.

An additional factor influencing the detection of deletions is the frequent presence of polyclonal infections in areas of moderate to high transmission. When parasite clones with intact *pfhrp2* genes coexist with others with deletions within the same infection, amplification techniques tend to favour the detection of intact copies. This can mask deletions and lead to an underestimation of their actual frequency. Conversely, poor DNA quality or low parasitaemia can cause amplification failures, which may be misinterpreted as deletions in the absence of appropriate internal controls.

Compliance with WHO recommendations for monitoring *pfhrp2* and *pfhrp3* gene deletions varied considerably among the studies included in this review. The WHO recommends adequate sampling and a standardised, stepwise molecular diagnostic process whereby only samples that are negative on HRP2-based rapid diagnostic tests (RDTs) but positive on microscopy or PfLDH-based RDTs should be considered deletion suspects, followed by molecular confirmation by PCR. In addition, confirmation of *P. falciparum* mono-infection and verification of DNA integrity using reference genes such as *msp1* and *msp2* are required before declaring a genetic deletion.

A major limitation across the literature is the absence of standardised methodologies and molecular assays for detecting *pfhrp2/3* gene deletions. In our review many studies fully complied with the criteria recommended by the WHO for confirming *pfhrp2/3* deletions 35/43(81%), 5/43(11.6%) were partially compliant, and 3(6%) were non-compliant. The lack of rigorous internal quality control measures to further reduce the risk of classification errors raises concerns about the reliability of some of the reported estimates

Marked heterogeneity was observed in DNA extraction procedures, diagnostic confirmation protocols, PCR approaches, sample sources, and study setting, undermining comparability across studies. Thus, part of the variability reported across studies may stem from differences in sampling strategies rather than genuine epidemiological variation in parasite populations. However, nested PCR appears to be the most used amplification technique. RT-qPCR, although effective, is less widely used, probably due to its cost or technical requirements.

This review also reveals a substantial delay between sample collection and the publication of study results, with a median lag of approximately three years. As diagnostic strategies face growing pressure to detect parasites carrying *pfhrp2/3* deletions, such delays may impede the development of timely, evidence based policy decisions.

To address this issue, reducing the interval between field sampling and data dissemination is crucial. Studies that minimise the time between data collection and publication are particularly important for guiding the rapid adoption of new diagnostic tools. Future surveillance systems should therefore emphasise rapid data sharing through mechanisms such as open access databases, preprint platforms, and real time molecular monitoring frameworks.

Further investigation is needed to understand how the prevalence of *pfhrp2/3* deletions evolves under the selective pressure exerted by HRP2 based rapid diagnostic tests.

## Conclusions

This systematic review highlights substantial spatial and methodological heterogeneity in the prevalence of *pfhrp2* and *pfhrp3* gene deletions and underscores their increasing impact for the accuracy of malaria diagnosis. The findings emphasise the need for diagnostic strategies that are adapted to regional epidemiological contexts, strengthened and harmonised molecular surveillance systems, and rapid dissemination of field data to reduce the risk of diagnostic failures in areas where deletions are emerging or highly prevalent. Strengthening standardised surveillance protocols and accelerating real-time reporting will be essential to maintain the reliability of malaria diagnostic tools and sustain progress toward malaria control and elimination in the context of ongoing parasite genetic evolution.
